# Digital Cueing With Laser Shoes Does Not Improve Walking in Parkinson’s Disease: Evidence Across Disease Severity and Freezing Status

**DOI:** 10.1177/15459683251369477

**Published:** 2025-09-11

**Authors:** Samuel Stuart, Rodrigo Vitorio, Lisa Graham, Julia Das, Richard Walker, Claire McDonald, Martina Mancini, Rosie Morris

**Affiliations:** 1 Department of Sport, Exercise and Rehabilitation, Northumbria University, Newcastle upon Tyne, UK; 2 Northumbria Healthcare NHS Foundation Trust, North Tyneside General Hospital, Newcastle upon Tyne, UK; 3 Gateshead Health NHS Foundation Trust, Gateshead, UK; 4 Department of Neurology, Oregon Health and Science University, Portland, Oregon, USA

**Keywords:** Parkinson’s disease, cueing, walking, freezing of gait, disease severity, sensors, rehabilitation

## Abstract

**Background:**

Gait impairment in Parkinson’s disease (PD) occurs early and pharmaceutical interventions do not fully restore this function. Visual cueing has been shown to improve gait and alleviate freezing of gait (FOG) in PD. Technological development of digital laser shoe visual cues now allows for visual cues to be used continuously when walking. This study aimed to investigate the effects of laser shoe visual cueing on gait in people with PD across different disease severity (i.e., Hoehn & Yahr [H&Y] stages I-III) and FOG status.

**Methods:**

Eighty people with PD (H&YI = 20, H&YII = 30 [15 FOG, 15 noFOG], H&YIII = 30 [15 FOG, 15 noFOG]) walked a 10 m straight path (back and forth) self-paced for 80 seconds without and then with laser shoe cues (participants were allowed 1-2 walks to familiarize with the cues). Inertial sensors were used to measure gait metrics. Laser cue line was set to usual step length for individuals based on their usual walk data from the inertial sensors.

**Results:**

Laser shoe cueing did not improve gait in PD regardless of disease severity or FOG status. Across all groups, participants decreased gait speed (*P* < .001), cadence (*P* < .001), arm range of motion (*P* < .005), and increased stride time, double support time (*P* < .001), elevation at midswing (*P* < .001), and gait variability (*P* < .001) with the laser shoes compared to usual walking.

**Conclusion:**

Digital laser shoe visual cues do not improve gait in people with PD across disease severity or FOG status. Further investigation is required to examine different cue settings or exposure periods.

## Introduction

Gait impairments caused by Parkinson’s disease (PD) are debilitating and challenging to treat.^1^ The most common gait impairments in PD include reduced speed and step/stride length, increased step-to-step variability, and freezing of gait (FOG) episodes^1^ (i.e., “brief, episodic absence or marked reduction of forward progression of the feet despite the intention to walk”^2^). With disease progression, gait impairments tend to worsen, and impairments increase the risk of falls and impact independence and overall quality of life in people with PD.^1^ Unfortunately, gait impairments only partially respond to antiparkinsonian medication.^1^ Therefore, rehabilitation interventions and cueing (i.e., internal or external prompts including auditory, visual or tactile modalities)^3-8^ are often used to improve gait in PD, particularly within the later stages of the disease (e.g., Hoehn and Yahr [H&Y] stages II-III).^9^ Indeed, previous studies have shown that cueing in early disease stages may impair gait further (e.g., increase gait variability) but may benefit gait in later stages of PD.^5^

The benefits of external visual cueing to gait in PD are well established.^10-12^ Visual cues can facilitate gait initiation, increase step/stride length, reduce step length variability, and alleviate FOG episodes in people with PD,^10-13^ but may not be useful for all gait issues (e.g., turning deficits).^14^ Traditionally, visual cues are implemented in the form of serial, spatially separated transverse stripes (i.e., tape or plastic to step on or over) on the floor (perpendicular to the direction of forward movement).^15-17^ When implementing visual cues in the home, they are often placed in walking areas that have been identified as problematic for people with PD, and are designed to reduce FOG or gait issues that lead to falls (e.g., scuffing the floor, transition points in flooring, or walking through doorways).^18^ Within clinical practice, the primary issue with traditional visual cue implementation is the stationary nature of the cues (i.e., they are fixed to the floor in one location of the home), which means that they cannot be used continuously throughout the day and people can tend to habituate (i.e., forget to use them) to the cues over time, as the novelty of the cues wears off with repeated use (e.g., the saliency of the cues reduces due to them consistently being in the visual environment). This limitation can be overcome by the implementation of visual cues through mobile digital health technologies or devices that allow the use of cues regardless of location and can present cues when needed.

In recent years, several mobile devices for implementing visual cues have been created (e.g., laser sticks, laser walkers),^19-21^ but the most recent development are laser shoes that do not require the use of hands and offer step-synchronized cueing.^19,20,22,23^ Laser shoes consist of normal shoes equipped with a transverse line-generating laser device mounted on the toe-area of the shoes^19^; the devices identify the persons walking pattern and, when the foot is on floor they shine a horizontal laser line in front of the contralateral foot to be stepped over or on. The intermittent nature of the laser shoe visual cues (i.e., the cue is only present when the foot is on the ground, and it only provides a cue for the next step) means that the cue may trigger more reflexive visual processing (i.e., reflexive eye movement to the cue/area of interest for the specific task). This may maintain novel saliency of the cue, which may alleviate the cognitive burden of walking and filtering the relevancy of areas in the visual environment, leading to better gait patterns.^16^ Preliminary evidence has suggested that laser shoe visual cueing may be able to alleviate FOG episodes in people with PD within a laboratory setting^19,22^ and home environment.^20^ However, it remains unclear what the impact of laser shoe visual cueing is on spatiotemporal gait parameters during continuous walking in people with PD across disease severity and gait impairment levels (e.g., FOG). This information is vital to understand if these interventions are to be used continuously in real-world settings. Therefore, this study aimed to investigate the immediate response to visual cueing with laser shoes on gait parameters during continuous walking in people with PD across different disease stages (i.e., H&Y stages I to III), and FOG groups (i.e., FOG vs noFOG). We hypothesized that visual cueing with laser shoes would improve spatiotemporal gait parameters in PD and may particularly benefit those with greater disease severity or who report FOG, in line with previous visual cueing studies in PD.^10,16,17,24-27^

## Methods

### Participants

Participants were recruited from Movement Disorder Clinics at Northumbria Healthcare NHS Foundation Trust and Gateshead Health NHS Foundation Trust and through the Parkinson’s UK research excellence network and the DeNDRoN Research Case Register. Eighty people with idiopathic PD were included in the study. Self-reported FOG was assessed with the New Freezing of Gait Questionnaire (NFOGQ).^28^ Subjects were categorized as “freezers” if they have experienced such a feeling or episode over the past month (i.e., score >1).

*Inclusion criteria*: Diagnosis of PD according to UK brain bank criteria; H&Y stage I-III; aged >50 years; able to walk and stand unaided; stable medication for the past 1 month and anticipated over a period of 6 months.

*Exclusion criteria*: Psychiatric co-morbidity; clinical diagnosis of dementia or other severe cognitive impairment); history of neurological disorders other than PD; acute lower back or lower extremity pain, peripheral neuropathy, rheumatic and orthopedic diseases; unable to comply with the testing protocol.

### Protocol

This study was carried out at the Clinical Gait Laboratory at Coach Lane Campus, Northumbria University, Newcastle upon Tyne. Study procedures were approved by the London-Bloomsbury NHS Research Ethics Committee (and Health Research Authority; 20/LO/1036, 05/10/2020), with written informed consent obtained prior to participation.^29^ Participants were tested in their “On” medication state (within ~60 minutes of anti-Parkinsonian medication intake).

### Clinical Assessment

Participant characteristics of disease duration, age, sex, weight, and height were recorded. Disease severity was assessed using the Movement Disorders Society (MDS-revised) Unified Parkinson’s Disease Rating Scale part III (MDS-UPDRS-III)^30^ and PD stage via Hoehn and Yahr Rating Scale (H&Y).^31^ Global cognitive function was assessed with the Montreal Cognitive Assessment (MoCA).^32^ Executive function was assessed using the Royall’s clock drawing (CLOX 1 & 2)^33^ and the Trail-making Test (TMT A & B).^34^ Fear of falling was assessed using the Falls Efficacy Scale—International version.^35^

### Gait Assessment

**Figure 1.**
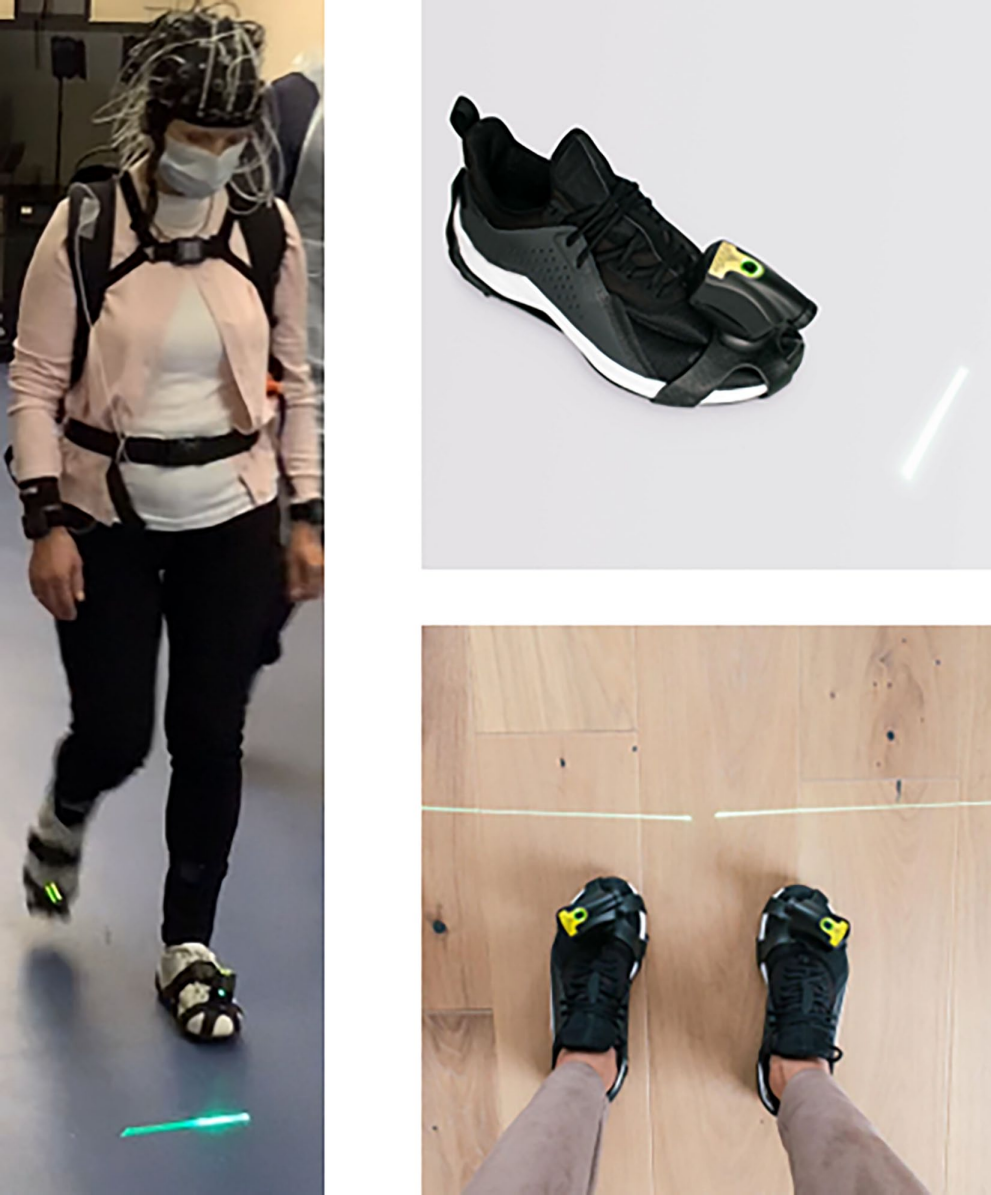
Laser shoe cueing device (path finder V2, walk with path, UK, https://www.walkwithpath.com/).

Participants were invited to walk, at self-selected comfortable pace, back and forth over a 10 m straight path (with a 180° turn at each end) for 80 seconds. Two conditions were tested, with a single trial for each condition: usual walk without cues (NoCue) and visual cueing with the laser shoes (Laser). The NoCue condition was performed first to ensure walking safety and minimize carryover effects from the cues. For the visual cueing condition, 2 laser devices (Figure 1, Path Finder version 2 (v2), Walk with Path, UK, https://www.walkwithpath.com/) were attached to participants shoes. Each laser device provided a visual cue (i.e., horizontal line) in front of the opposite foot, with the participant instructed to step over the line. The devices were adjusted to a usual step length for the participant (based on wearable sensor data from the NoCue condition) with participants comfortably able to step over the lines while walking. Participants were allowed to familiarize themselves with the cues (1-2 10 m walks) before assessment.

Eight inertial measurement units (Opal v2, APDM, USA) (IMU; combined accelerometer, gyroscope, and magnetometer) were used to quantify spatiotemporal gait parameters at a sampling rate of 128 Hz. They were located at the sternum and lower back, and bilaterally on the wrists, shanks, and feet. Each IMU was securely fixed to the participant’s body with Velcro straps. Spatiotemporal gait measures were calculated using the Mobility Lab software (Mobility Lab v2, APDM, USA). The following gait measures were calculated: gait speed, stride length, stride time, cadence, arm range of motion, foot strike angle, double support phase (% of gait cycle), and gait variability (i.e., standard deviation of consecutive steps/strides).^36^ The number of steps obtained for gait variability was 54.6 ± 15.0 steps for NoCue and 48.7 ± 12.2 steps for Laser cue walking.

### Statistical Analysis

Statistical analysis was undertaken using SPSS version 26 (IBM, USA). All statistical tests were carried out with an alpha of *P* < .05 level of significance. Normality of data was checked with box plots and Kolmogorov-Smirnov tests. Demographics were reported across disease severity groups (H&YI-III). Dependent on the normality of data, one-way analysis of variance (ANOVA) or Kruskal-Wallis tests were used to compare demographic and clinical variables across groups, according to data distribution. Chi-Square test was used to compare gender across groups. Bonferroni post hoc analysis was performed to provide specific group comparison information.

To examine the influence of laser shoe visual cueing on gait in PD across disease severity stages a linear-mixed effects model (LMEM) was used to compare gait outcomes across walking conditions (i.e., NoCue vs Laser) and groups (i.e., H&YI, H&YII, H&YIII), while controlling for FOG status. A separate LMEM was used to compare gait outcomes across conditions with FOG status groups (i.e., FOG vs noFOG). Gait outcomes with non-normal distribution were transformed (log or inverse transformation method) before entering the LMEMs.

## Results

### Participants

Participants had mild to moderate disease severity and mostly preserved cognition, as measured with the MoCA (Table 1). ANOVA/Kruskal-Wallis test revealed significant group differences for MDS-UPDRS III score and disease duration (*P* < .001, i.e., H&I < H&YII < H&YIII). Further, H&YII and H&YIII groups had greater levodopa equivalent daily dose than H&YI (*P*=.001). These group differences were expected as participants were distributed into groups according to H&Y stage. Groups were matched for all other cognitive/demographic variables (Table 1).

**Table 1.** Participants Characteristics.

Variable	H&Y I	H&Y II	H&Y III	*P*	Bonferroni Post hoc
FOG status	−FOG = 20/+FOG = 0	−FOG = 15/+FOG = 15	−FOG = 15/+FOG = 15	—	—
Male/female	M = 15/F = 5	M = 20/F = 10	M = 22/F = 8	.775	—
Age (years)	67.5 ± 7.5	68.7 ± 6.7	72.2 ± 9.7	.141	—
Height (cm)	168.8 ± 8.8	166 ± 21.4	167.7 ± 9.1	.939	—
Body mass (kg)	78.7 ± 11.8	85.9 ± 22.9	79 ± 15.2	.784	—
Years of education	12.5 ± 2.6	14.5 ± 3.4	12.9 ± 2.9	.068	—
Disease duration (years)	2.2 ± 1.9	5.5 ± 4.5	7.5 ± 5.7	<.001*	H&YI < H&YII < H&YIII
MDS-UPDRS III (score)	17.4 ± 7.5	38.1 ± 11.2	49.4 ± 12.7	<.001*	H&YI < H&YII < H&YIII
LEDD (mg/day)	314 ± 270.3	662.4 ± 418.6	706.1 ± 382.9	.001*	H&YI < H&YII-III
NFOG-Q (score)	0 ± 0	7.2 ± 8.7	7.5 ± 8.7	<.001*	H&YI < H&YII-III
MoCA (score)	27.4 ± 2.8	27.5 ± 1.9	25.9 ± 2.6	.030*	ns
Trail Making Test A (sec)	42.8 ± 38.3	38.7 ± 13.8	47 ± 25.6	.144	—
Trail Making Test B (sec)	102 ± 81.9	90.9 ± 59.7	127 ± 87.8	.093	—
Trail Making Test B-A (sec)	59.2 ± 54.2	52.1 ± 50.4	80 ± 66.8	.091	—
CLOX 1 (score)	12.8 ± 1.2	12.8 ± 1.3	12.1 ± 1.7	.194	—
CLOX 2 (score)	13.5 ± 1	13.3 ± 1.3	12.9 ± 1.4	.199	—

Abbreviations: FOG, freezing of gait; H&Y, Hoehn & Yahr Rating Scale; LEDD, Levodopa equivalent daily dose; MDS-UPDRS, Movement Disorders Society-Unified Parkinson’s Disease Rating Scale; MoCA, Montreal Cognitive Assessment; NFOG-Q, New Freezing of Gait Questionnaire; ns, non-significant.

* *P* < .05.

### No Significant Gait Changes With Laser Shoes Across Disease Severity

LMEMs controlled for FOG status, revealed no significant interactions between walking condition and disease stage (group) for gait outcomes (all *P* > .05, Table 2), indicating that H&Y stage does not influence gait response to cueing with the laser shoes in people with PD in H&YI to H&YIII.

**Table 2.** Impact of Laser Shoes on Gait Characteristics in Parkinson’s Disease Across Disease Severity and Freezing Status.

Outcomes	H&YI		H&YII		H&YIII		H&Y × Walking condition^a^	NoFOG		FOG		FOG × walking condition	PD		All PD walking condition (NOcue vs Laser)^a^	
	NOCue	Laser	NOCue	Laser	NOCue	Laser	*F, P*	NOCue	Laser	NOCue	Laser	*F, P*	NOCue	Laser	*F, P*	Comparisons
Speed	0.89 (0.17)	0.78 (0.24)	0.93 (0.14)	0.83 (0.20)	0.78 (0.16)	0.66 (0.23)	0.226, .798	0.87 (0.16)	0.76 (0.23)	0.85 (0.18)	0.75 (0.24)	0.238, .627	0.87 (0.17)	0.76 (0.23)	40.499, <.001*	NOcue > Laser
Cadence	100.24 (8.64)	85.92 (14.51)	104.97 (10.77)	92.05 (14.91)	98.85 (10.86)	82.11 (16.61)	0.934, .398	101.64 (9.27)	86.72 (14.01)	101.46 (12.50)	87.85 (18.81)	0.079, .779	101.57 (10.56)	87.14 (15.85)	140.990, <.001*	NOcue > Laser
Stride length	1.05 (0.16)	1.07 (0.19)	1.06 (0.14)	1.07 (0.16)	0.94 (0.17)	0.94 (0.20)	0.247, .782	1.03 (0.16)	1.03 (0.20)	1.00 (0.18)	1.01 (0.19)	0.081, .777	1.02 (0.17)	1.03 (0.19)	0.418, .520	—
Stride time	1.21 (0.10)	1.44 (0.26)	1.16 (0.12)	1.34 (0.20)	1.23 (0.14)	1.52 (0.32)	0.962, .387	1.19 (0.11)	1.43 (0.24)	1.20 (0.16)	1.43 (0.33)	0.084, .773	1.20 (0.19)	1.43 (0.27)	141.486, <.001*	NOcue < Laser
Step time	0.61 (0.05)	0.72 (0.13)	0.58 (0.06)	0.67 (0.10)	0.62 (0.07)	0.76 (0.16)	2.080, .132	0.60 (0.06)	0.71 (0.12)	0.60 (0.08)	0.72 (0.16)	0.030, .864	0.60 (0.64)	0.72 (0.13)	141.244, <.001*	NOcue < Laser
Arm range of motion	22.60 (9.64)	18.02 (10.15)	24.32 (11.49)	19.45 (13.63)	24.91 (14.11)	21.44 (15.22)	0.046, .955	23.37 (11.33)	17.58 (10.88)	25.27 (13.10)	23.55 (16.26)	2.745, .102	24.11 (12.00)	19.80 (13.36)	26.924, <.001*	NOcue > Laser
Foot strike angle	19.49 (5.21)	19.54 (4.12)	19.27 (5.33)	19.60 (5.95)	16.10 (6.28)	17.29 (6.27)	0.631, .535	18.10 (5.73)	18.77 (5.35)	18.30 (6.03)	18.72 (6.35)	0.000, .987	18.18 (5.81)	18.76 (5.70)	1.668, .200	—
Double support phase	22.90 (2.84)	25.88 (9.21)	23.13 (2.77)	24.85 (3.29)	25.43 (4.46)	27.69 (6.09)	0.147, .863	23.43 (3.42)	26.11 (6.63)	24.66 (3.91)	26.17 (5.77)	1.266, .264	23.91 (3.64)	26.13 (6.29)	19.996, <.001*	NOcue < Laser
Elevation at midswing	1.68 (0.54)	2.41 (1.06)	1.90 (1.58)	2.57 (1.80)	1.73 (0.94)	2.15 (1.07)	0.477, .822	1.60 (0.63)	2.18 (0.88)	2.07 (1.68)	2.71 (1.95)	0.022, .884	1.78 (1.17)	2.38 (1.39)	89.577, <.001*	NOcue < Laser
Stride length SD	0.06 (0.04)	0.07 (0.03)	0.07 (0.04)	0.07 (0.03)	0.06 (0.02)	0.07 (0.02)	0.457, .635	0.07 (0.04)	0.07 (0.03)	0.06 (0.03)	0.07 (0.04)	0.031, .860	0.07 (0.04)	0.07 (0.03)	3.428, .068	—
Stride time SD	0.04 (0.02)	0.08 (0.06)	0.04 (0.01)	0.07 (0.04)	0.05 (0.02)	0.10 (0.06)	0.169, .845	0.04 (0.02)	0.08 (0.05)	0.05 (0.02)	0.09 (0.06)	0.178, .674	0.04 (0.18)	0.08 (0.05)	80.327, <.001*	NOcue < Laser
Step time SD	0.03 (0.01)	0.05 (0.04)	0.02 (0.01)	0.04 (0.02)	0.03 (0.01)	0.06 (0.03)	0.522, .596	0.03 (0.01)	0.05 (0.03)	0.03 (0.01)	0.07 (0.04)	0.170, .681	0.03 (0.01)	0.05 (0.03)	66.702, <.001*	NOcue < Laser
Arm range of motion SD	4.37 (1.93)	4.96 (3.28)	5.01 (2.48)	5.44 (3.89)	4.84 (2.42)	6.27 (6.15)	0.043, .957	4.45 (1.94)	5.05 (4.04)	5.33 (2.77)	6.57 (5.54)	0.526, .470	4.79 (2.32)	5.62 (4.68)	0.167, .684	—
Foot strike angle SD	2.78 (1.21)	2.76 (0.92)	3.23 (1.33)	3.01 (1.14)	2.47 (0.61)	2.86 (0.80)	1.522, .225	2.81 (1.18)	2.81 (0.89)	2.89 (1.05)	3.02 (1.08)	0.047, .828	2.84 (1.12)	2.89 (0.96)	0.342, .561	—
Double support phase SD	1.41 (0.38)	2.37 (1.93)	1.47 (0.35)	1.93 (0.70)	1.63 (0.55)	2.44 (1.14)	0.995, .374	1.47 (0.44)	2.17 (1.36)	1.58 (0.45)	2.31 (1.13)	0.059, .809	1.51 (0.44)	2.23 (1.27)	43.527, <.001*	NOcue < Laser
Elevation at midswing SD	0.46 (0.10)	0.58 (0.18)	0.51 (0.31)	0.59 (0.27)	0.48 (0.14)	0.60 (0.21)	0.026, .975	0.45 (0.11)	0.55 (0.15)	0.54 (0.31)	0.67 (0.20)	0.044, .835	0.48 (0.21)	0.59 (0.23)	40.374, <.001*	NOcue < Laser

Abbreviation: NOcue: walking without cues.

* *P* < .05.

a Indicates control for FOG status in statistical analysis.

### No Gait Changes With Laser Shoes in People With Freezing of Gait

A separate LMEM revealed no significant difference between walking with or without laser shoes in freezers or non-freezers for gait outcomes (all *P* > .05, Table 2), indicating that FOG status (freezer vs non-freezer) does not influence gait response to laser shoe cueing in people with PD.

### Overall Gait Changes With Laser Shoes in Parkinson’s Disease

**Figure 2.**
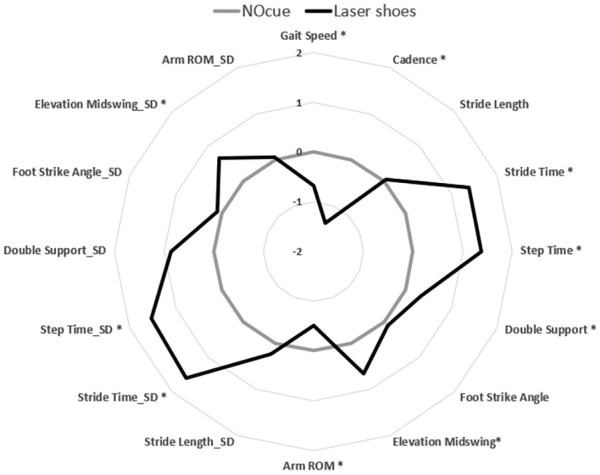
Radar plot representation of gait changes (*z*-score) from baseline (NOcue) to visual cueing with laser shoes.

Laser shoes significantly impacted several gait outcomes in people with PD (Table 2). Compared to walking with no cues, participants decreased gait speed (*P* < .001), cadence (*P* < .001), arm range of motion (*P* < 0.001), and increased stride time (*P* < .001), double support phase (*P* < .001), elevation at midswing (*P* < .001), stride time variability (*P* < .001), step time variability (*P* < .001), double support phase variability (*P* < .001), elevation at midswing variability(*P* < .001) while walking with the laser shoes (Figure 2). This may indicate a more cautious but variable gait pattern being undertaken when walking with the laser shoe visual cues in PD. Surprisingly, stride length did not significantly change with laser shoe visual cueing in PD (Table 2).

## Discussion

This study investigated the effects of visual cueing with laser shoes on spatiotemporal gait parameters in people with PD across disease severity (H&YI to H&YIII), and FOG status. Surprisingly, findings do not support our hypothesis that visual cueing with laser shoes would promote immediate benefits to spatiotemporal gait parameters in PD, particularly in those with greater disease severity or FOG. Our findings showed that when walking with the laser shoes people with PD had reduced gait speed, cadence, arm range of motion and increased stride time, double support phase, elevation at midswing, and gait variability, regardless of disease stage or FOG status. Results suggest that visual cueing with laser shoes does not improve gait in people with PD and may even worsen some gait characteristics, which requires further investigation and cautious clinical application.

### Laser Shoe Cueing Impacts Gait in PD

Laser shoes did not significantly improve gait characteristics in PD across different disease severity and FOG status groups. There are two theories on the changes seen with the laser shoe cueing in PD; (1) people with PD adopted a more cautious gait approach with the laser shoe cueing; and (2) that laser shoe cueing impaired gait in PD.

The changes in gait characteristics from usual walking to walking with laser shoe cueing across the full PD cohort may suggest a more cautious gait approach as they slowed down (i.e., reduced speed, stride/step time etc.) and picked their feet up more (i.e., increased midswing elevation) because of stepping over the cued laser lines, which has been seen in previous research.^22^ The changes in gait with laser shoe cueing suggest a predominant executive-attentional control of movement, as gait changes observed with the laser shoes are similar to those observed during dual-task walking, which is a walking condition that requires increased use of executive-attentional resources.^37-39^ Furthermore, slowness of movement, large variability, and limited degrees of freedom (e.g., reduced arm range of motion) are commonly observed at the initial stage of motor learning (i.e., the cognitive stage).^40^ It is possible that short-term (within session) use of the laser shoe cues triggered this initial motor learning process in those with PD, and that longer-term use of the laser shoe cues may be needed to establish learning to improve gait characteristics. However, while participants may have implemented a more cautious approach when walking with the laser shoe cues the approach may have placed them at increased risk of falls, as the additional cognitive resources required by the laser cues and the increased variability of their gait pattern may lead to inattention to the environment (i.e., focus only on the laser line directly in front of them) and inability to accurately respond to hazards.^41-44^

It could alternatively be suggested that gait worsened with laser shoe cueing in those with PD, which is a simple explanation for the changes in gait characteristics seen. For example, reduced gait speed and increased gait variability are considered to be undesirable gait characteristics which are linked to increased falls risk in PD.^41-44^ Interestingly, stride length did not significantly change with laser shoe visual cueing, which is surprising considering the target of visual cueing is often on the stepping process (e.g., step or stride length improvement). However, previous studies have shown similar findings in non-laser visual cueing (i.e., transverse taped lines to step over set to usual step length or above) with reduced speed and little change in stride length,^16^ unless cues are set to greater than usual stride length.^12,17^ However, unlike the laser shoe cues, gait variability is usually improved with non-laser visual cues due to the repetitive nature of the reciprocal stepping over the lines (i.e., multiple transverse lines on floor at set distance to step over or on),^17^ which is different from the intermittent laser line presentation from the laser shoes. Increased gait variability may be derived from the intermittent appearance of the laser line with each step and only presenting one line for the next step introduces the need to process visual and motor information continuously during walking (with limited cognitive resources to do so), which may improve reflexive visual response (i.e., looking at the line on the floor without conscious thought due to the reflexive saccadic eye movement to the appearance of the laser line target) but does not allow for optic flow improvement,^24,45^ or a set motor program with fixed step distance or plan for multiple stepping targets to be implemented from the start of the walk.^16^ For example, the laser shoe cues require online real-time processing and implementation of sensory and motor programs with limited executive-attentional resources, whereas usual visual cueing may free up online cognitive resources through allowing a set motor program to be implemented from the beginning of the walk.^16^ Therefore, gait may have worsened in PD with the laser shoe cueing due to the additional requirement to step over the laser cue maxing out the limited cognitive (executive-attentional) resources available to those with PD, which is like dual-tasking (i.e., the laser shoe cues become a dual-task). This is vital to understand in future research as dual-task protocols are used within laboratory settings as a proxy to investigate how a person may behave in the real-world where their attention will not solely be on their walking task, and therefore in the real-world the laser shoe cues may be an additional task that would place the person at risk of poorer walking performance and potentially falls.

Ultimately, there may be a combination of the two theories at play, but it is difficult to understand the exact reasons for the gait changes with laser shoe cues as there is no “gold-standard” cueing modality (e.g., auditory, visual, tactile) or set of cue settings (e.g., set to step length, greater than step length, intermittently shown or constantly visible, worn on the person or set in the environment) to compare gait outcomes across in PD. In general, findings from this study suggest that gait is impacted by the laser shoe cues, and at the very least the specific cue modality should be tested with patients under clinical supervision to ensure that they are not at greater risk of gait impairment or falls when using the laser shoe cues.

### Future Directions for Cueing in PD

Cueing for gait impairment (or FOG) in PD is a complex intervention that has poorly understood underlying mechanisms and therefore the application of cues within clinical practice is often variable or sub-optimal. Several studies have alluded to aspects of the underlying mechanisms involved in cue response in PD, such as attentional or motor cortical resources being required which may differ with FOG status,^6,12^ but a comprehensive understanding of cue response is lacking. As a result, within clinical practice a trial-and-error method is often implemented, with clinicians working with patients to test out different cueing modalities (auditory, tactile, or visual) and settings (e.g., different tempos, device placement, line distance), but this is time consuming and often only has short-term effect (i.e., response may habituate over time or modalities/settings may need to be changed).^8^ The limitations of current clinically used cueing methods, such as metronomes or taped lines, have paved the way for modern technology to potentially provide a greater range of cue modalities or settings, which may enable greater benefit by more patients for longer periods.

There is now a plethora of digital therapeutic cueing devices aimed at gait (or FOG) improvement for people with PD available from various developing or established companies (e.g., Path Finder from Walk with Path, CUE1 from Charco Neurotech, NexStride from De Oro, Tempo from GaitQ, Stroll augmented reality etc.). These cueing devices are primarily to be used independently by patients within real-world settings (e.g., at home, in community), but systematic evaluation of these cueing technologies within real-world settings is lacking. Furthermore, from an early stage (i.e., concept stage) co-design of the digital intervention with patients and relevant stakeholders or experts needs to take place so that developed solutions are not only effective, but also practical (and unobtrusive) for use within the real-world (i.e., companies may make a device, but if people with PD will not use it then it is pointless).^46,47^ For example, laser shoe visual cues require a relatively obtrusive device to be placed on the feet, which is visible to other people, whereas patients may prefer visual cueing that is only visible to themselves (e.g., visual cue glasses,^48^ or future augmented reality through future glasses that look like typical glasses rather than huge obtrusive headsets).^49-51^ Future cue development requires thorough understanding of not just the scientific or therapeutic aspects of the intervention, but also the usability and preference for individual patients.

Despite the promise of digital cueing methods in PD, there remains no single or combination of devices that can improve gait across all patients in all situations, and those that may be able to assist a patient at one timepoint may not continue to do so long-term.^52^ Digital cueing methods have improved the delivery of cueing with a wider range of settings available (e.g., tempo, saliency, color, volume) and potential to use in different locations. Many digital cueing devices also have regulatory clearances for use as medical devices within healthcare settings, for example Path Finder laser shoes are CE marked and have been recommended by the National Institute of Clinical Excellence (NICE) for alleviation of FOG in PD,^53^ which is despite very limited evidence available for this and the current study findings of impact (or worsening) on gait in PD. However, the core problem of understanding the reason why people with PD do or do not respond to different cues and whether this changes at different disease stages or with selective gait issues (e.g., FOG) remains unknown, and therefore developing a digital cueing technology that is universally effective is incredibly difficult. Future studies need to investigate the real-time (online) neural activity occurring when walking with various cueing modalities with various settings in large samples of those with PD, across disease stage and gait impairment (e.g., FOG, early or late disease stages) severities.^29^ This will allow more targeted and specific cueing interventions to be developed that may be personalized to individuals, which may need to change in modality or settings overtime to ensure continued effect with disease progression.

### Study Limitations

The present study has several limitations. First, our interest was in gait characteristic response to laser shoe visual cues in PD, and therefore we did not objectively or subjectively measure FOG episodes during walking. Future evidence is needed to generate objective (i.e., digital data or device measured) data on FOG response to laser shoe visual cues in real-world settings (i.e., where FOG mostly occurs), such as novel algorithms for FOG ratio detection,^54^ to support the previous subjective video evaluations previously performed in laboratory settings.^19,20^ Second, only the immediate effects of laser shoe visual cueing that was set to a usual step length for individual participants were investigated. Future studies should investigate whether more practice is required for gait benefits to be achieved with the cues, or whether setting the visual cue laser line to greater than usual step length is more beneficial to gait in PD. Thirdly, we did not ask patients to feedback on the usability or perception of the laser shoe cues, which needs to be further investigated to understand if patients would use the laser shoe device.

## Conclusion

This was the largest study of laser shoe digital visual cueing that has been undertaken in people with PD. Current findings suggest that visual cueing with laser shoes does not improve gait parameters in people with PD across different disease severities and FOG status. Laser shoe visual cues should be cautiously implemented in clinical practice (or by patients), as increased gait variability seen with laser shoe cueing may place people with PD at greater risk of falls. Further research is required to fully understand the underlying reasons for gait characteristic changes in PD with digital cueing devices before routine implementation within clinical practice.
